# Plant Polyphenol Pyrogallol and Polyamine-Based Co-Deposition for High-Efficiency Nanofiltration Membrane Preparation towards Inorganic Salt Removal

**DOI:** 10.3390/membranes12111151

**Published:** 2022-11-16

**Authors:** Jiawen Wu, Zhiwen Li, Qingfeng Zhou, Mercy Chigwidi, Yang Jiao, Yanchao Xu, Hongjun Lin

**Affiliations:** College of Geography and Environmental Sciences, Zhejiang Normal University, Jinhua 321004, China

**Keywords:** nanofiltration, membrane separation, co-deposition, polyphenols, amines

## Abstract

The co-deposition between polyphenols and amines has been demonstrated in order to prepare positively charged nanofiltration (NF) membranes for multivalent cation rejection in recent years; however, the low reactivities of the involved polyphenols usually cause a long co-deposition time and unsatisfactory rejection. Herein, a novel plant polyphenol (PG) was co-deposited with tetraethylenepentamine (TEPA) in a much shorter time period to prepare positively charged NF with high multivalent cation rejection membranes. The performance of the co-deposition membranes can be easily controlled by adjusting the mass ratio of PG and TEPA, reaction time, and pH value of the buffer solution. The optimal membrane, prepared under a polyphenol and polyamine mass ratio of 1:1, coating time of 2 h, and pH value of 8.0, shows a decent pure water permeability of 8.43 L m^−2^ h^−1^ bar^−1^ while maintaining a superior 96.24% MgCl_2_ rejection. More importantly, the universality of this method was corroborated by employing other amines with different molecular weights in the co-deposition. This work provides new insights for the preparation of high-performance positively charged NF membranes.

## 1. Introduction

Membrane-based separation technology has emerged as a promising alternative to conventional technologies, such as adsorption [1,2], extraction [3,4], and advanced oxidation [5,6], for water treatment, as it can realize the fractionation of small solutes from liquids with low energy and few byproducts. Nanofiltration, a typical pressure-driven membrane separation process, is able to separate divalent and multivalent ions from water, and therefore plays a crucial role in the desalination application [7]. So far, many approaches, including interfacial polymerization (IP) [8,9] and co-deposition [10,11], have been used for the preparation of NF membranes.

A typical IP process includes immersing an ultrafiltration (UF) substrate in an amine aqueous solution and an acid chloride n-hexane solution successively, to generate a polyamide selective layer on the substrate top surface. The prepared polyamide NF membrane is negatively charged due to the hydrolysis of the excessive acyl chlorides to carboxylic acid at membrane surface. This means the polyamide membrane has undesirable selectivity to cations due to the weak Donnan effect [12]. In addition, the difficult multistep operations during the IP process also hinder the preparation of a defect-free and high-performance NF membrane.

The co-deposition of plant polyphenols and polyamines, inspired by the self-polymerization of dopamine to form polydopamine coatings, has attracted extensive research interests in recent years [13,14]. Polyphenols widely exist in plant tissues and have the advantage of much lower cost than dopamine. It has been reported that the catechol groups in polyphenols can react with amine groups and form a co-deposition layer on any substrate surface. So far, several polyphenols, such as catechol [15], tannic acid (TA) [16], and epigallocatechin gallate (EGCg) [17], have been used with polyamines to form co-deposition layers on UF substrates to prepare NF membranes. Due to the existence of amine groups at the co-deposition layer surface, these NF membranes are usually positively charged, and show great potential in separating cations from water. However, these works usually suffer from some drawbacks. For example, a long co-deposition time ranging from 4 to 12 h is usually needed [15,16,17,18,19,20,21], which is undesirable from the viewpoint of membrane preparation. In addition, the prepared membranes show unsatisfactory divalent cation rejections lower than 85%. To rapidly prepare a polyphenol and polyamine co-deposited NF membrane with high divalent cation rejection is of great importance in the NF field, but still a tough challenge.

In this study, a novel plant pyrogallol (PG) was co-deposited with various amines on polyacrylonitrile (PAN) UF substrates to prepare NF membranes. Unlike previously reported catechol, TA, and EGCg co-deposition, the co-deposition between PG and amines after a relatively short time of 2 h gives rise to a superior 96.24% MgCl_2_ rejection. The effect of mass ratio between PG and amines, co-deposition time, buffer solution pH, and amine type on the NF membrane morphology, surface properties, and separation performance were systematically investigated.

## 2. Materials and Methods

### 2.1. Materials

Polyacrylonitrile (PAN, ≥96%, molecular weight: 75,000 g mol^−1^) was purchased from Shanghai Petrochemical Co., Ltd. (Shanghai, China). Polyethylene glycol 800 (PEG-800, ≥99%) was obtained from Xilong Chemical Industrial Co., Ltd. (Zhongshan, China). 1-Methyl-2-pyrrolidinone (NMP, ≥98%), ethanol (≥99.5%), n-hexane (≥97%), and hydrochloric acid (HCl, 36.0~38.0%) were bought from Sinopharm Chemical Reagent Co., Ltd. (Shanghai, China). Pyrogallol (PG, ≥99%), tetraethylenepentamine (TEPA, ≥90%), diethylenetriamine (DETA, ≥99%), polyethyleneimine 600 (PEI-600, ≥99%), and polyethyleneimine 10000 (PEI-10000, ≥99%) were received from Aladdin Industrial Corporation (Shanghai, China). Ethylenediamine (EDA, ≥99%), magnesium chloride (MgCl_2_, ≥98%), calcium chloride (CaCl_2_, ≥96%), magnesium sulfate (MgSO_4_ ≥ 98%), sodium sulfate (Na_2_SO_4_, ≥98%), and sodium chloride (NaCl, ≥99.5%) were purchased from Jinhua Southeast Chemical Instrument Co., Ltd. (Jinhua, China). Tris(hydroxymethyl)aminoethane (Tris, Molecular Weight: 121.14 g mol^−1^) was bought from VWR, Part of Avantor Chemicals, LLC (Shanghai, China). Deionized water was prepared by Pure water manufacturing apparatus, produced by Merck & Co. Inc. (Rahway, NJ, USA).

### 2.2. Preparation of PAN UF Membrane

Firstly, 19 g PAN and 1 g PEG-800 powders were dissolved in 80 g NMP, and stirred in a 60 °C water bath for 24 h until the solutes were completely dissolved. After that, the casting solution was then stood in a 60 °C water bath for 12 h for degassing. The PAN membranes were obtained by the traditional phase inversion method, and the specific method can be found in our previous works [22,23]. After that, the PAN membranes were soaked in fresh deionized water at 4 °C to prevent bacterial growth on the membrane surface.

### 2.3. Preparation of PG/Polyamine NF Membrane

**Figure 1:**
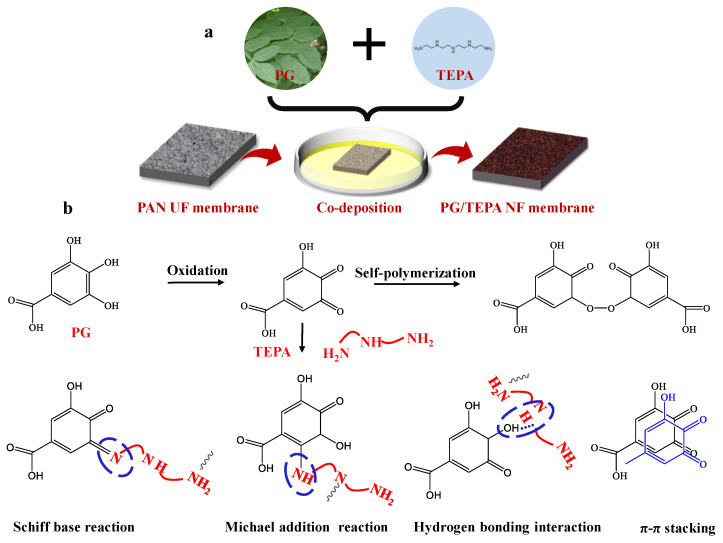
(**a**) Preparation process of PG/TEPA NF membrane and (**b**) possible reaction mechanism between PG and TEPA.

To prepare the coating solution, the PG was dissolved in the designed pH value Tris-HCl buffer solution first, and TEPA was then dissolved in the above solution. The PAN membrane was immersed in the coating solution to prepare the PG/TEPA NF membrane. For example, the PAN membrane was soaked in coating solution (PG/TEPA = 1:1, the concentration of PG was 1.0 mg/mL, pH = 8.0) and shaken for 2 h to prepare the optimal PG/TEPA NF membrane. The specific preparation process and the possible reaction mechanism are shown in Figure 1.

### 2.4. Characterizations of PAN Membrane and PG/TEPA Membranes

A scanning electron microscope (S-4800, Tokyo, Japan) was used to observe the surface and cross-sectional morphology of the membrane. For the cross-section morphologies, the dry membrane samples were required for brittle fracture by liquid nitrogen. The chemical structure of the membrane was identified by attenuated total reflectance Fourier transform infrared spectroscopy (ATR-FTIR, NEXUS 670, Waltham, MA, USA) with collected spectra in the range of 400–4000 cm^−1^. A SurPASS electrokinetic analyzer (Anton Paar, Graz, Austria) was used to measure the ζ potential of all membranes at pH values from 3 to 10. The water contact angle (WCA), which determines the hydrophilia of the PG/TEPA membrane, was measured with a contact angle meter (Kino Co., Ltd., New York, NY, USA). All membranes were dried in an oven at 40 °C for 12 h to ensure dryness before characterization measurements.

### 2.5. Evaluation of Membrane Performances

The separation performances of the various membranes were evaluated at 5 bar and 25 °C, using a cross-flow device (the cross-flow rate was 100 mL/min) with an effective filtration area of 22.1 cm^2^. All membranes were pre-compacted with deionized water at 5 bar for 1 h to obtain a stable PWP. Experiments were performed with feeding solutions of MgCl_2_, CaCl_2_, MgSO_4_, Na_2_SO_4_, and NaCl at concentrations of 1000 mg/L. Each membrane was tested at least 3 times. The PWP of membrane was calculated using Equation (1), as follows:

(1) $$P=\frac{V}{\Delta P\times A\times t}$$

where *P* (L m^−2^ h^−1^ bar^−1^) is the permeation flux; *V* (L) is the feeding solution volume; *t* (h) is the operation time; *A* (m^2^) is the membrane effective filtration area; Δ*P* (bar) is the filtration pressure.

The formula of rejections is given in Equation (2):

(2) $$R=\left(1-\frac{C_{p}}{C_{f}}\right)\times 100\%$$

where *R* is the rejection; *C_p_* is the concentration in filtrate; *C_f_* is the concentration in the feed solution. The inorganic salt concentrations above were determined by an electrical conductivity meter (Leici DOS-307A, Shanghai, China). For long-term stability testing, the PG/TEPA NF membrane was tested continuously for 48 h, with PWP and rejection measurements taken every 6 h.

## 3. Result and Discussion

### 3.1. Morphologies of PAN Membrane and PG/TEPA Membrane

**Figure 2:**
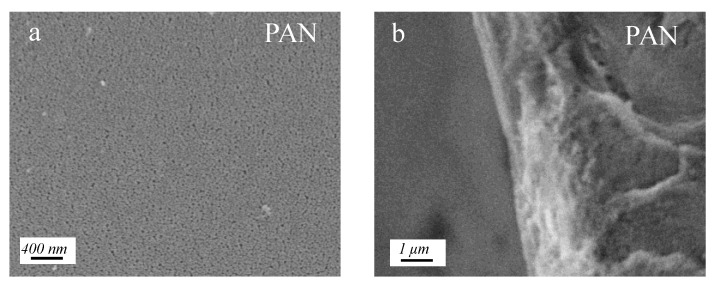
SEM images of PAN UF membrane: (**a**) surface morphology and (**b**) cross-section morphology of PAN UF membrane.

**Figure 3:**
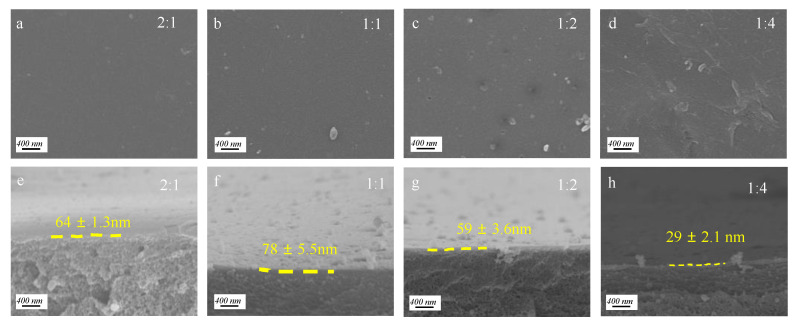
Surface SEM images of PG/TEPA NF membranes with PG to TEPA mass ratios of (**a**) 2:1, (**b**) 1:1, (**c**) 1:2, and (**d**) 1:4; cross-section SEM images of PG/TEPA NF membranes with PG to TEPA mass ratios of (**e**) 2:1, (**f**) 1:1, (**g**) 1:2, and (**h**) 1:4. (The effect of different ratios on the membrane morphology is investigated when the constant reaction time is 5 h and the pH is 8.5).

The morphologies of membranes were characterized by SEM. As shown in Figure 2, the pristine PAN UF substrate exhibits micropores of around 20 nm diameter and a relatively smooth surface. By contrast, the surface of the PG and TEPA co-deposited membrane surfaces show no obvious pores (Figure 3), which suggests the construction of a co-deposition layer on the substrate surface. Similar to the other polyphenols, such as tannic acid and catechol, PG can also be rapidly oxidized to a quinone structure. These highly reactive quinone intermediates can further undergo self-polymerization, or interact with amine groups in TEPA by covalent bonds generated via Michael addition. These covalent bonds, together with other non-covalent bonds, such as hydrogen bonds, π-π stacking and charge transfer interactions, contribute to the formation of a dense co-deposition layers on the substrate surface [21,24]. Figure 3 shows the surfaces and cross-sectional morphologies of the PG and TEPA co-deposited membranes at different PG/TEPA mass ratios. When the mass ratio of PG/TEPA is 2:1, the thickness of the co-deposition layer is ~64 nm. Upon the ratio of PG/TEPA reaches 1:1, the thickness of the co-deposition layer increased to ~78 nm. At the same time, some PG/TEPA aggregates grew on the membrane surface because of the enhanced deposition behavior at a higher monomer. However, further increasing the mass ratio 1:4 results in a decrease in the co-deposition layer thickness from 78 nm to 29 nm due to the fact that too many amine groups would depress the non-covalent bonds during the co-deposition process, and therefore inhibit the co-deposition onto the substrate surface [25].

**Figure 4:**
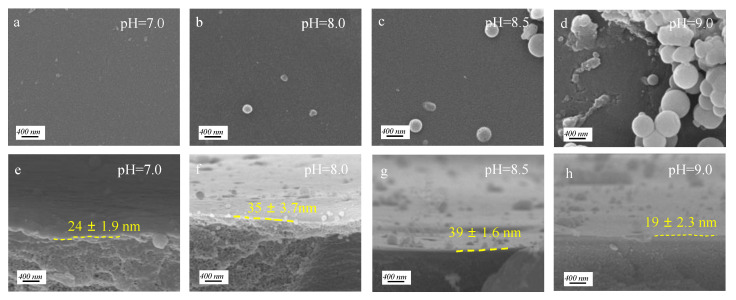
Surface SEM images of PG/TEPA NF membranes at (**a**) pH 7.0, (**b**) pH 8.0, (**c**) pH 8.5, (**d**) pH 9.0; cross-section SEM images of PG/TEPA NF membranes at (**e**) pH 7.0, (**f**) pH 8.0, (**g**) pH 8.5, (**h**) pH 9.0. (The effect of different pH on the membrane morphology is investigated under the condition of constant PG/TEPA = 1:1 and reaction time of 2 h.).

The effect of buffer pH values on membrane morphology was further investigated, and the results were shown in Figure 4. Under a pH of 7.0, a smooth membrane surface was obtained (Figure 4a), while as the pH value gradually increased to 9.0, the membrane surface exhibits a large number of nano-aggregates due to the fact that weak alkaline buffer solution is beneficial to the oxidation and further self-polymerization of PG (Figure 4b–d), and therefore facilitates the covalent bonding between aromatic rings to generate nano-aggregates. The thickness of the co-deposition layer is also affected by the pH value of the buffer solution. At pH = 8.0 and pH = 8.5, the top layer thickness is ~35 nm, higher than that of 22 nm at pH = 7.0 (Figure 4e–g).

### 3.2. Chemical Characterization of Membranes

**Figure 5:**
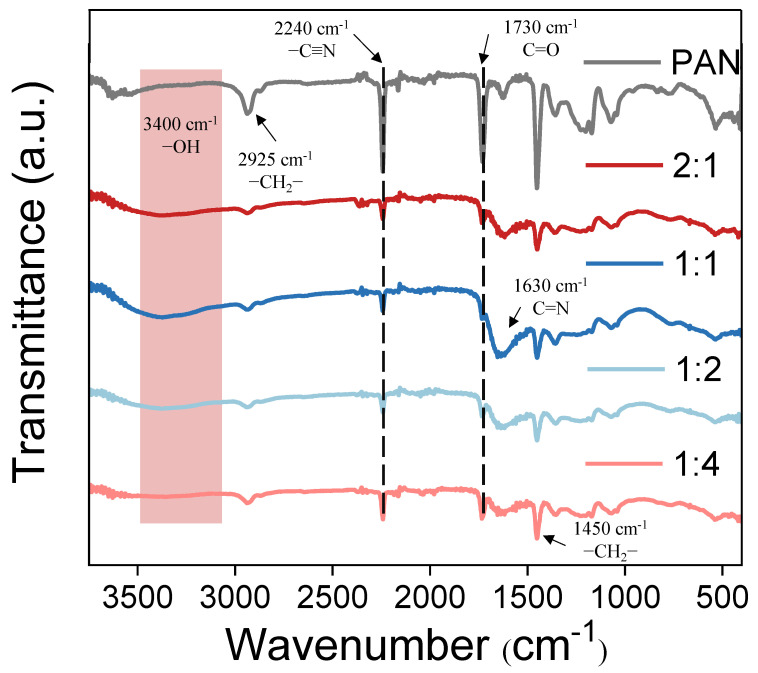
FT−IR spectrum of PAN UF membrane and PG/TEPA NF membranes with different PG and TEPA mass ratios. (The effect of different ratios on the chemical properties of the membrane is investigated under the condition that the reaction time is 2 h and the pH is 8.5).

The chemical characterizations of the PAN substrate and co-deposited membranes were evaluated by the ATR-FTIR spectra. The pristine PAN substrate exists characteristic peaks at 2240 cm^−1^, 2925 cm^−1^, and 1450 cm^−1^ (Figure 5), corresponding to the stretching vibration of the –C≡N and the stretching and bending vibrations of –CH_2_^−^, respectively. An additional absorption peak at 1730 cm^−1^ is due to the stretching vibration of C=O because of the hydrolysis of –C≡N [26,27]. Then, intensities of the above peaks are weakened in the spectra of the co-deposited membranes; meanwhile, some new peaks emerge at around 3400 cm^−1^ and 1630 cm^−1^ due to O–H stretching vibration and C=N stretching vibration relating to polyphenols and amines, respectively [28,29]. These indicate the successful formation of a co-deposition layer on the surface of the PAN substrate [30]. Upon the mass ratio of PG/TEPA reaches to 1:1, these two peaks have the strongest intensities, which is consistent with the thickest co-deposition layer at this mass ratio, as disclosed by the SEM image in Figure 3f. However, further increase in the PG/TEPA mass ratio suppresses the co-deposition process and leads to a decrease in the intensities of the above characteristic peaks [18,21,24,31].

### 3.3. Surface Properties of Membranes

**Figure 6:**
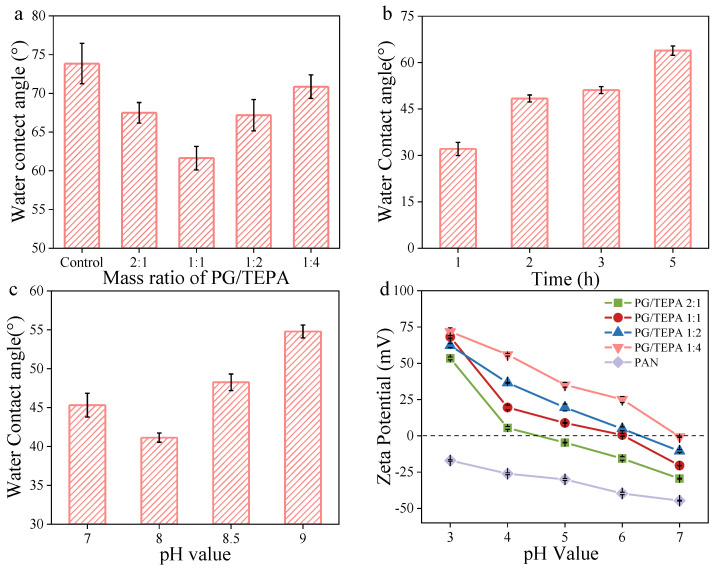
Surface properties of PG/TEPA membrane: (**a**) water contact angles of PG/TEPA membranes with different mass ratios of PG/TEPA (control means the PAN UF membrane, the effect of different ratios on WCA is explored under the 2 h reaction time and pH 8.5); (**b**) water contact angles of the PG/TEPA membranes with different reaction times (the effect of reaction times on WAC is explored with a constant PG/TEPA ratio of 1:1 and a pH of 8.5); (**c**) pH values of buffer solutions (the effect of pH on WCA is explored with a constant PG/TEPA = 1:1 and a reaction time of 2 h); and (**d**) zeta potential of PAN UF membrane and PG/TEPA NF membranes with different PG and TEPA mass ratios (the effect of different ratios on ζ potential is explored with a reaction time of 2 h and the pH of 8.5).

The water contact angle (WCA) reflects the hydrophobic/hydrophilic properties of the membrane surface, and the WCA of membranes are further measured to evaluate the effect of co-deposition on membrane surface properties [17]. The pristine PAN membrane shows a WCA of 73.9°, and the co-deposited membranes exhibit lower WCAs due to the hydrophilic groups at the co-deposition layer. The WCA first decreased, then increased with increasing PG/TEPA mass ratio from 2:1 to 1:4, and the lowest WCA of 61.6° is obtained at a PG/TEPA mass ratio of 1:1. This can be rational considering the thickest co-deposition formed at this mass ratio, as revealed by SEM in Figure 3h. The WCA increased from 32.1° to 63.8° with the prolonging of the reaction time from 1 h to 5 h (Figure 6b) due to the reduction in phenolic hydroxyl groups on the membrane surface, resulting in a decrease in hydrophilicity [32]. The contact angle at pH 8.0 is the smallest because PG and TEPA react most actively under this weak alkaline condition [33] (Figure 6c). Since the maximum deposition of PG and TEPA occurs at pH = 8, the minimum WCA is 41.1°. The zeta potential of the PAN UF membrane is negatively charged in the pH range of 3 to 7. The zeta potentials of PG/TEPA membranes, as shown in the Figure 6d, gradually increase with increasing TEPA ratio in solution, and most of co-deposited membranes are positively charged under a general NF operation pH of 6 [34]. The zeta potential of the PG/TEPA NF membrane rises after co-deposition (Figure 6d) due to the protonation of the amine group at the co-deposition layer [19,35].

### 3.4. Nanofiltration Performance of Membranes

**Figure 7:**
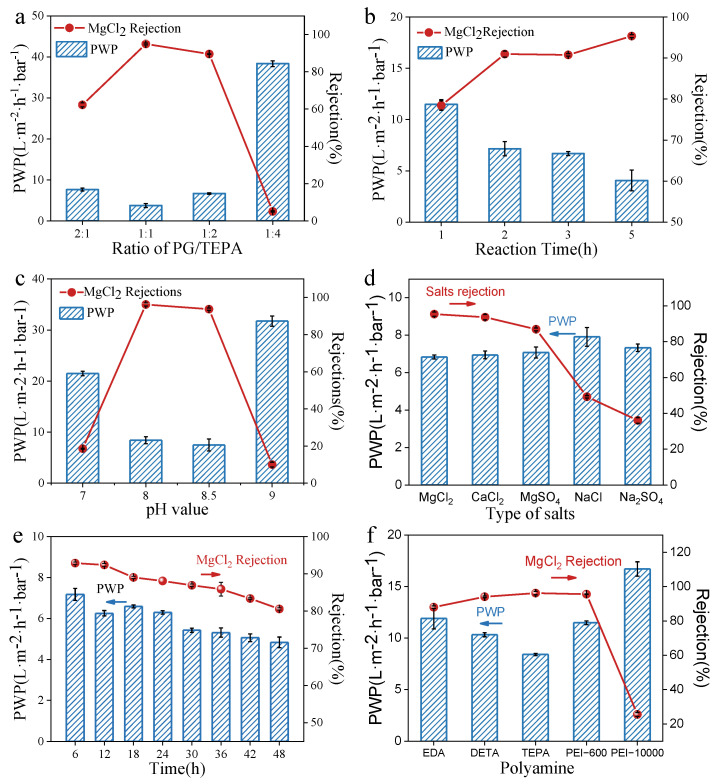
Separation performances of the various PG/TEPA membranes with different (**a**) ratios of PG to TEPA (5 h, pH 8.5), (**b**) reaction times (PG/TEPA = 1:1, pH 8.5), and (**c**) pH values of buffer solutions (PG/TEPA = 1:1, 2 h). (**d**) Ion screening performances of the PG/TEPA membrane (optimal condition: PG/TEPA = 1:1, 2 h, pH 8.0) for different inorganic salt solutions with the concentration of 1000 mg/L. (**e**) Long-term separation performances of the PG/TEPA membrane (optimal condition: PG/TEPA = 1:1, 2 h, pH 8.0) within 48 h. (**f**) Separation performances of the PG/polyamine membranes (optimal condition: PG/polyamines = 1:1, 2 h, pH 8.0), the feeding solution is MgCl_2_ solution at a concentration of 1000 mg/L.

The effect of the PG/TEPA mass ratio on the separation performance of the co-deposition membrane is shown in Figure 7a. The rejection of the PG/TEPA membrane for MgCl_2_ is affected by both size sieving and the Donnan effect, and the PG/TEPA membrane prepared at a PG/TEPA mass ratio of 1:1 shows the highest MgCl_2_ rejection of 94.94%, and a PWP of 3.74 L·m^−2^·h^−1^·bar^−1^. Therefore, in the following experiments, the mass ratio of PG to TEPA was fixed at 1:1, and other conditions were further optimized. With prolonging the co-deposition time from 1 h to 5 h, the rejection of MgCl_2_ increased from 78.43% to 95.36%, at the same time, PWP continually decreased from 11.48 L m^−2^ h^−1^ bar^−1^ to 4.06 L m^−2^ h^−1^ bar^−1^ (Figure 7c), because the thickness of the deposition layer increases with the co-deposition time. It was further proved that, as shown in Figure 7c, a weak alkaline condition at pH 8.0 rendered the PG/TEPA membrane a commendable PWP of 8.43 L m^−2^ h^−1^ bar^−1^ and a high MgCl_2_ rejection of 96.24%.

The rejections of the optimal PG/TEPA membrane for MgCl_2_, CaCl_2_, MgSO_4_, NaCl, and Na_2_SO_4_ are 95.4%, 93.7%, 87.0%, 49.3%, 36.1%, respectively (Figure 7d). The rejection of divalent cations (Mg^2+^, Ca^2+^) by PG/TEPA membrane is much higher than that of monovalent sodium salts, which is due to the Donnan exclusion. The long-term separation performance of the optimal membrane within 48 h was shown in Figure 7e. The PWP decreases slightly on account of membrane compaction, while the MgCl_2_ rejection also decreases, which might be due to the layer instability under long-term high pressure and cross-flow conditions [34].

**Figure 8:**
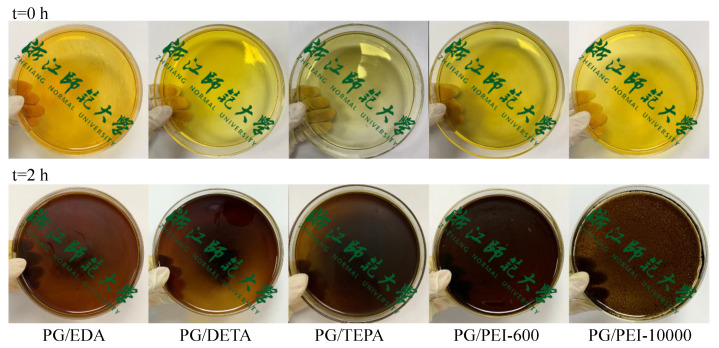
Optical images of PG/polyamine coating solutions before and after a 2 h co-deposition process (preparation conditions: PG/polyamine = 1:1, pH 8.0).

Amines with different molecular weights were employed to illustrate the universality of co-deposition. As shown in Figure 7f, the MgCl_2_ rejections of PG/EDA, PG/DETA, PG/TEPA, and PG/PEI-600 co-deposited membranes are 88.03%, 94.13%, 96.24%, and 95.61%, respectively. Since long-chain amines provide more reaction sites to react with PG to form thicker selective layers, the molecular weight of the amine functional group increases, and the rejection increases [16]. While further increasing the molecular weight of amines results in the formation of a large number of precipitates in the buffer solution instead of co-deposition on the PAN membrane, the separation performance of the PG/PEI-10000 membrane drops drastically, as demonstrated by the optical images of the co-deposition solutions in Figure 8.

Table 1 lists the separation performances of some previously reported PDA- or polyphenol-coated NF membranes and the PG/TEPA-PAN NF membrane in this study. It can be found that the PG/TEPA-PAN membrane shows a higher MgCl_2_ rejection than the other membranes. Furthermore, our membrane exhibits an overwhelming performance compared to the CCh [18,36], TA [37], and PDA [38] -modified membranes in both water permeance and MgCl_2_ rejection.

## 4. Conclusions

Herein, the co-deposition between PG and TEPA on a PAN substrate was proposed to prepare a high-performance NF membrane in a short time period for divalent cation removal. It was proven that the co-deposition time, pH of the buffer solution, and mass ratio of PG/TEPA are important preparation parameters for improving the performance of the co-deposition membrane. The optimized membrane shows good separation performances toward various divalent cations, which has practical and economic benefits in desalination. The universality of this strategy was demonstrated by the co-deposition of polyamines of different molecular weights with PG. The structural stability of the co-deposited membrane will be improved in our further work. This work introduces a new method for creating and modifying NF membranes, and expands the application of NF membranes in the treatment of inorganic salt wastewater.

## Figures and Tables

**Table 1:** Comparison of the separation performances of various polyphenol and polyamine NF membranes.

Membrane *	Deposition Time (h)	Active Layer Thickness (nm)	Testing Time (h)	PWP (L m^−2^ h^−1^ bar^−1^)	MgCl_2_ Feed Concentration (mg/L)	MgCl_2_ Rejection (%)	Refs.
CCh/PEI-PAN	6	130	2	2.60	2000	85.20	[15]
TA/DETA-PAN	12	450	1	4.50	2000	83.50	[16]
EGCg/PEI-PES	6	55	0.5	8.60	1000	33.00	[17]
CCh/PEI/GA-PSF	4	500	1	4.17	1000	88.00	[18]
(TA/DETA/Ag)-PAN	5	135	2	5.36	2000	86.5	[19]
PDA-CuSO_4_/H_2_O_2_-PAN	12	105	2	10	1000	52	[20]
PDA/GNPs/PEI-PAN	6	125	2	11	1000	90	[21]
PG/TEPA-PAN	2	35	1	8.43	1000	96.24	This work

* PDA, PEI, EGCg, GA, CCh, PSF, TA, GNPs, PDA, PES are the abbreviations of polydopamine, polyethylenimine, epigallocatechin gallate, glutaraldehyde, catechol, polysulfone, tannic acid, gold nanoparticles, polydopamine, polyethersulfone, respectively.

## Data Availability

Not applicable.
